# Recurrent Hepatitis in Two Iranian Children: A Novel (Q166R) Mutation in EIF2AK3 Leading to Wolcott-Rallison Syndrome

**DOI:** 10.5812/hepatmon.10124

**Published:** 2013-06-09

**Authors:** Babak Behnam, Marjan Shakiba, Ali Ahani, Maryam Razzaghy Azar

**Affiliations:** 1 Cellular and Molecular Research Center, Iran University of Medical Sciences, Tehran, IR Iran; 2 Department of Medical Genetics and Molecular Biology, Faculty of Medicine, Iran University of Medical Sciences, Tehran, IR Iran; 3 Ali-Asghar Children Hospital, Iran University of Medical Sciences, Tehran, IR Iran; 4 Department of Pediatrics, Mofid Hospital, Shahid Beheshti University of Medical Sciences, Tehran, IR Iran; 5 Department of Genetics and Reproduction, Avicenna Research Center, Tehran, IR Iran; 6 Endocrine and Metabolic Research Center, Tehran University of Medical Sciences, Tehran, IR Iran

**Keywords:** Wolcott-Rallison Syndrome, Diabetes Mellitus, Spondyloepiphyseal Dysplasia

## Abstract

Early-onset diabetes, liver dysfunction, growth retardation, spondyloepiphyseal dysplasia, and tendency to skeletal fractures due to osteopenia are characteristics of Wolcott-Rallison syndrome (WRS). Eukaryotic translation initiation factor 2α kinase (EIF2AK3) is the only known gene, which is responsible for this rare autosomal recessive disorder. Here, we report two siblings a girl and a boy with diabetes mellitus (DM) who presented in one and two months of age respectively. Recurrent self-limiting hepatitis developed later, and severe hepatic failure resulted in death of the first child. The second child visited was a 7.75 year old boy who had spondyloepiphyseal dysplasia and subclinical hypothyroidism besides DM and recurrent hepatitis. We suggested WRS for this patient, and it was confirmed by identification of a novel homozygous missense mutation (Q166R) in exon 3 of the EIF2AK3 gene. The aim of this report is to remind the possibility of WRS in isolated neonatal diabetes; while, the other clinical manifestations of this syndrome including its major symptom of recurrent hepatitis may appear later.

## 1. Introduction

Wolcott-Rallison syndrome (WRS) (OMIM 226980) is a rare disorder but has been reported as the most common known genetic cause of permanent neonatal diabetes mellitus (PNDM) which occurs in consanguineous pedigrees (1, 2). Other common genetic causes of PNDM are proposed as a mutation in KCNJ11 (NM_000525), ABCC8 (NM_000352.2), and INS (NM_000207). WRS, as a monogenic (single gene) autosomal recessive disorder is caused by mutations in *EIF2AK3* gene. WRS is characterized by infancy permanent diabetes, hepatic and renal dysfunction which are mostly recurrent, self-limiting, and episodic. It is accompanied by multiple epiphyseal or spondyloepiphyseal dysplasia, osteoporosis, growth retardation, and congenital heart anomaly (2). Liver dysfunction sometimes leads to hepatic failure, which is mostly accompanied by acute multiorgan failure (encephalopathy, renal, and bone marrow failure) and may result in the patient’s death. However, hepatic and renal functions returned to normal in survived patients. Unfortunately, Genetic counseling and analysis for this condition is usually delayed until WRS is fully presented. *EIF2AK3* is a type I transmembrane protein which functions as an endoplasmic reticulum (ER) stress sensor for protein synthesis regulation via phosphorylating the α-subunit of the eukaryotic initiation factor-2 (eIF2) in the ER (3, 4). Therefore, diabetes may be considered as an ER stress disease (5).

## 2. Case Presentation

This study was performed in the Molecular Genetics Laboratory of Ali-Asghar Children's Hospital of Iran University of Medical Sciences, in accordance with the Declaration of Helsinki. An informed consent was obtained from the parents on behalf of the child. Clinical information was obtained using a standardized questionnaire from outpatient and hospital records. Parents were first cousin. Since the first child was died, a single proband was tested for *EIF2AK3 *mutation with a suspected diagnosis of Wolcott-Rallison Syndrome based on early-onset diabetes (within the first 15 months of age), unexplained liver dysfunction, and skeletal dysplasia. The Figures of changes in liver enzymes were drawn by IBM SPSS 19.

### 2.1. *EIF2AK3* Gene Analysis

Genomic DNA was extracted from peripheral leukocytes using standard method. The coding exons and the intron-exon boundaries of the *EIF2AK3* gene were amplified via PCR; primers and conditions were available upon request. Single-strand sequencing was performed utilizing gene specific primers and standard methods on an ABI 3730 (Applied Biosystems, Macrogen, South Korea). Sequences of all amplicons were compared with the published template (accession no. AF110146.1) using Mutation Surveyor (version 3.20; SoftGenetics, State College, PA). Any changes in the sequence were checked against published polymorphisms and mutations and for conservation across species.

### 2.2. Patients

#### 2.2.1. Patient 1

**Figure 1.**
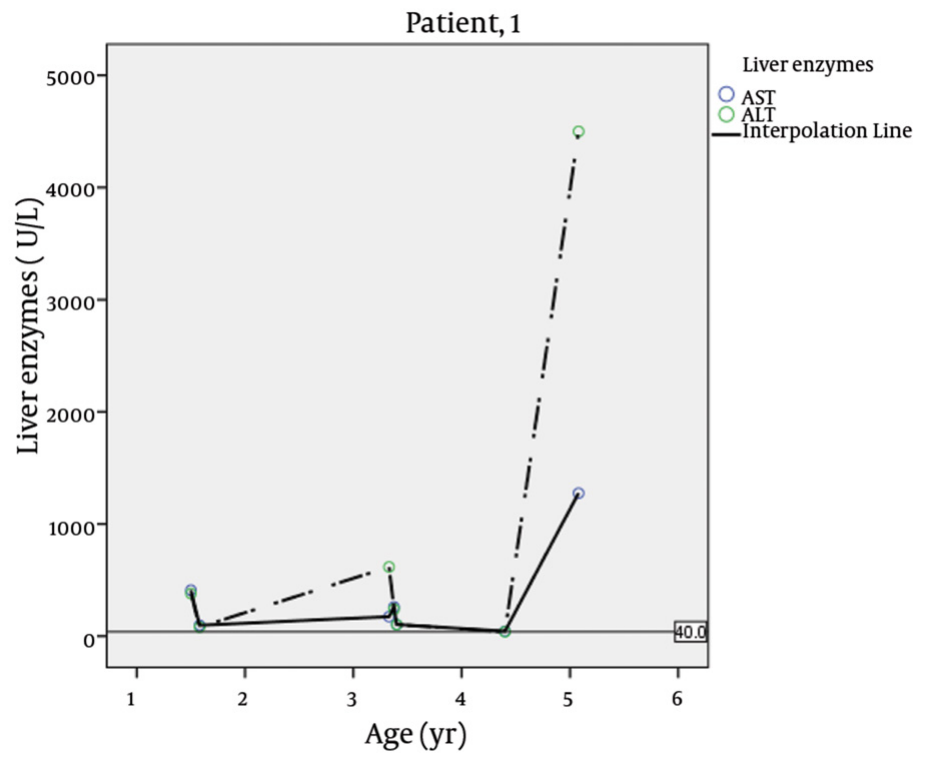
Liver Enzymes Fluctuations in Different Ages of Patient 1 (Dead)

The first patient was a girl, the first child of healthy parents with consanguineous marriage. She had diabetes since 40 days of age and atrial septal defect (ASD) which improved spontaneously. She had frequent hospital admissions due to vomiting, abdominal pain and distention, acholic stool and generalized edema. Hepatomegaly and ascites were reported in her medical records. Laboratory tests showed abnormal liver function. The liver enzymes in different ages are illustrated in Table 1 and Figure 1. Other tests were total bilirubin, 3 mg/dL (normal range, 0 - 1.2); direct bilirubin, 1.9 mg/dL (0 - 0.3); Alkaline Phosphatase, 1720 IU/L (180 - 1200); viral hepatitis markers had negative results.

 Blood urea nitrogen, creatinine, serum electrolytes, calcium, phosphorus, lipid profile, and protein electrophoresis had normal findings. Anti-insulin autoantibody anti-islet cell antibody had negative results, while anti-glutamic acid decarboxylase (anti GAD) was 19.6 IU/mL (positive, > 12). Recurrent episodes of hepatitis had usually occurred concomitant with viral upper respiratory infections. Abnormal walking had begun when she was 3 years old, and the patient eventually died with hepatic failure at 5 years of age.

**Table 1.** Liver Enzymes in Different Ages in Two Patients

Patients	Age, y	AST^a^,U/L, Ref. Value 15 - 45	ALT,U/L, Ref. Value 15 – 45
**Patient 1**	1.5	410	380
	**1.58**	97	81
	**0.91**	1200	1300
	**3.33**	174	617
	**3.38**	261	240
	**3.4**	103	103
	**4.4**	44	39
	**5.08**	1275	4500
**Patient 2**	1.5	48	144
	**1.58**	48	144
	**4.58**	51	25
	**4.75**	31	25
	**4.91**	31	25
	**5.16**	40	23
	**7.16**	140	510
	**7.25**	56	193

^a^Abbreviations: ALT, alanine aminotransferase; AST, aspartate aminotransferase

#### 2.2.2. Patient 2

**Figure 2.**
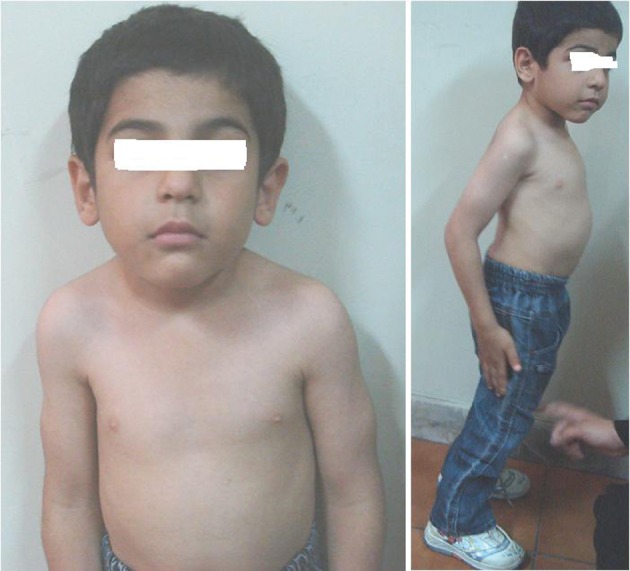
Photograph of the Patient 2 Demonstrating Posture

**Figure 3.**
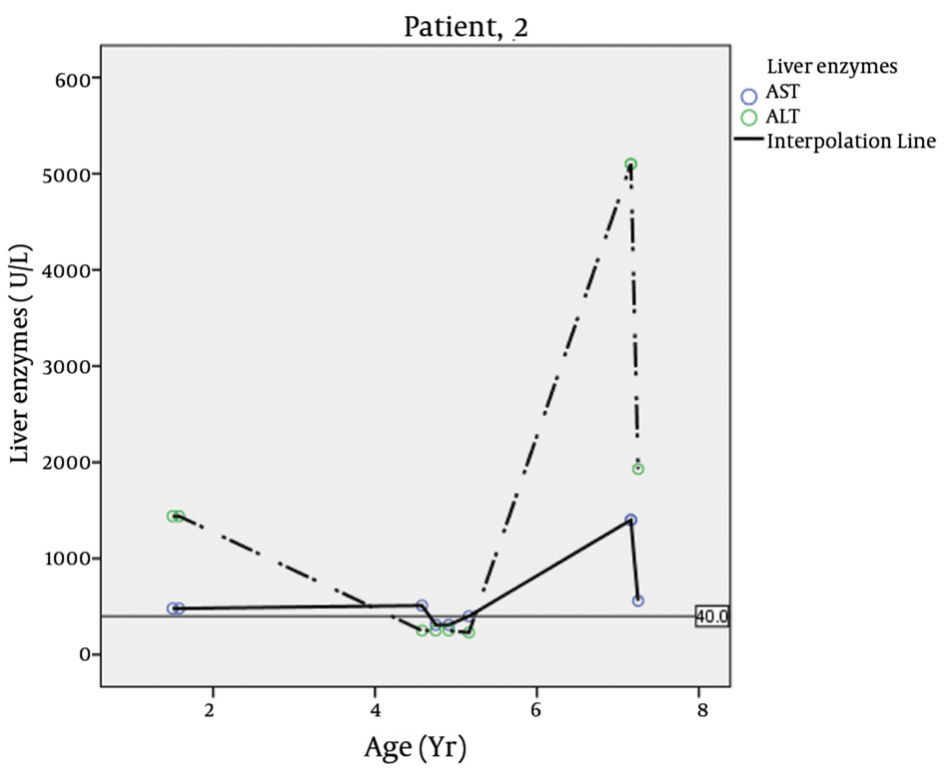
Liver Enzymes Fluctuations in Different Ages of Patient # 2

The second patient was the brother of the first child who was visited by us at 4.4 years of age (Figure 2). He was born full term by caesarean section with a birth weight of 2.4 Kg (< 5th percentile) which was considered small for gestational age. Neonatal diabetes had been diagnosed at 2 months of age when he presented with poor feeding and dehydration. Treatment with regular and NPH insulin was started for him twice daily upon diagnosis. He had the history of hospital admission at 11 months of age due to vomiting and abdominal distention accompanied by generalized edema. Laboratory tests showed abnormal liver function with highly elevated liver enzymes, anemia with neutropenia, and lymphocytosis at that age (Table 2). Fluctuations in liver enzymes are seen in different ages (Table 1, Figure 3).

**Table 2.** Laboratory Tests of Patient # 2

Laboratory Tests	Patient Values	Reference Values
**Total bilirubin, mg/dL**	0.5	0-1.2
**Direct Bilirubin, mg/dL**	0.1	0-0.3
**Alkaline Phosphatase, IU/L**	1021	180-1200
**White Blood Cell Count, K/MCL**	5.5	4.3-10.8
**Hemoglobin, g/dL**	10.8	11.2-14.1
**Platelets, K/MCL**	323	150-410
**Neutrophils, % of Leukocytes**	16	35-80
**Lymphocytes, % of Leukocytes**	81	40-60
**Bun, mg/dL**	16.4	5-20
**Cr, mg/dL**	0.8	0.5-1.2
**Hemoglobin A1c, %**	11.7	4.8-5.9
**PT, s**	13	13
**PTT, s**	35	35-45
**Lactate dehydrogenase, U/L**	1137	Up to 500
**Creatinine phosphokinase, U/L**	155	24-195
**Ammonia, μmol/L**	18.8	10-47
**Lactate, mg/dL**	11.6	5-20

The report of abdominal ultrasonography confirmed hepatomegaly, mild pericholecystic edema, normal spleen, and enlarged hyper echoic kidneys but no bright liver. Doppler sonography of hepatic vessels and portal system had normal findings. The clinical signs were improved spontaneously; AST and ALT returned to normal level but recurred after viral upper respiratory infections. He has been under treatment with levothyroxine sodium due to subclinical hypothyroidism (T4: 7.9 µg/dl, TSH: 7.35 µIU/L), since one year ago.

**Figure 4.**
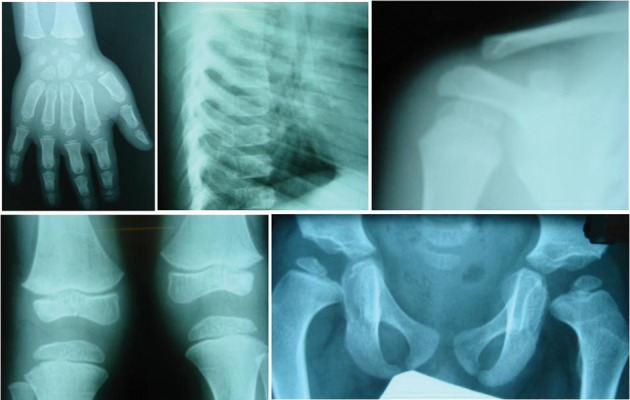
Radiographic Findings: X-ray Films Demonstrating Beaking of Thoracic Vertebrae, Generalized Epiphyseal Dysplasia and Osteopenia of the Ribs, Hand, Knees, and Pelvis (Skeletal System)

**Figure 5.**
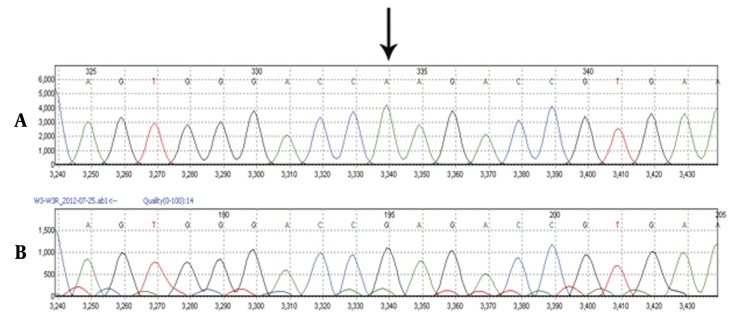
Electrophoregrams Illustrates A) Normal, B) Abnormal sequences; The arrow shows the site of mutation in the patient

Serum cortisol at 8 AM was 27.6 µg/dL. Other laboratory tests like blood gas, lipid profile, immunoglobulin A had normal results, and endomysial IgA and antitissue transglutaminase had negative findings. Alpha 1 antitrypsin was 2.94 g/L (0.7 – 1.7). He had difficulty in walking since three years of age. Bone survey reported small and irregular epiphysis compatible with multiple epiphyseal dysplasias (Figure 4). We proposed Wolcott-Rallison syndrome as the most possible diagnosis for this patient with respect to clinical manifestations, paraclinical findings, and family history, which led us to perform genetic study to confirm the diagnosis. Sequencing analysis showed that the patient is homozygous for a novel missense mutation, Q166R (c.604A > G; p.Glu166R), in exon 3 of the *EIF2AK3* gene (Figure 5). Her mother was heterozygous carrier for this mutation.

## 3. Conclusions

In this study, clinical diagnosis of Wolcott-Rallison Syndrome (WRS) was confirmed by the identification of a novel homozygous missense mutation (Q166R) in exon 3 of the *EIF2AK3* gene. WRS has been reported as the most common known genetic cause of permanent neonatal diabetes mellitus (PNDM) in consanguineous pedigrees (1). Wolcott-Rallison syndrome is a rare autosomal recessive disease, first reported in 1972 by Wolcott and Rallison who described three siblings with infantile onset diabetes mellitus and multiple epiphyseal dysplasias (2). Liver dysfunction, renal insufficiency, organomegaly, joint stiffness, hypoplastic pancreas, cardiac and neurodevelopmental defects, and central hypothyroidism were added to the list of problems in later reports (6-10). Renal insufficiency was not seen in our two patients. The gene responsible for this disorder was determined in year 2000 based on genetic studies of two consanguineous families as *EIF2AK3* (or PEK) which encodes translation of the pancreatic eukaryotic initiation factor 2α (eIF2α) kinase (11). Senee et al studied twelve families with 18 cases of WRS in 2004, while 17 patients of 18 carried *EIF2AK3* mutations resulting in a truncated or missense version of the protein (12). In a study *EIF2AK3* was sequenced in 34 probands with infancy-onset diabetes with clinical phenotype suggestive of WRS and homozygous or compound heterozygous for mutation of *EIF2AK3* were found in 25 probands (1). Although the mutation which we have found in this study (Q166R) is a missense mutation, and does not result in the protein truncation or frame shift, it may have a great impact on the protein function leading to WRS. This might be probably due to making an amino acid change in N-terminus of the protein compared to some other mutations reported in much downstream and C-terminal part of the protein. For instance, there are some recent reports of nonsense mutations (e.g. R491X, C532X, c.1408_1409insT, Gln398Stop, p.Pro285AlafsX3) in exons 5, 7, 8, and 9 of the *EIF2AK3* gene leading to protein truncation, and therefore WRS (13-16). Although it is not reported in Eif2ak3 knockout mice, phenotypic features including severity and unexplained hypoglycaemic episodes are suggestive of hepatic impairment and renal failure (17). It has been shown that *EIF2AK3* gene and its product are developmentally required in formation and function of the insulin-secreting β-cells during fetal life as well as early neonatal period (3). Recently, a complex bi-directional link between the diabetes and liver has been highlighted, in particular addressing nonalcoholic fatty liver disease (NAFLD) and the roles of endoplasmic reticulum (ER) and lipotoxicity in its etiopathogenesis (18). Moreover, the association of diabetes with NAFLD as an inter-related pathogenic mechanism has been recently shown (19). It is shown that steatohepatitis may be induced by ER stress, and some genes including osteocalcin and PNPLA3/adiponutrin are proposed to have major role in this regard. Studies have shown that ER stress is reversed by Osteocalcin through nuclear factor-kappaB signaling, so impaired insulin sensitivity resulted from diet-Induced obesity improves (20, 21). In general, ER stress as a recently studied mechanism of NAFLD is interesting while intervention strategies targeting ER dysfunction may provide hope for future therapy and prevention in hepatic dysfunction state as well as diabetes. In our patients hepatic dysfunction was deteriorated by upper respiratory infections which may cause ER stress. Although mitochondrial beta-oxidation defects may cause liver failure, but these disorders are accompanied by clinical and laboratory manifestations that were not found in our patients, like hypoglycemia, high creatinine phosphokinase, high lactate, myopathy and some others. Any infant with diabetes mellitus should be followed for the signs and symptoms of WRS while all findings of this syndrome might be obscured at the time of diagnosis of isolated neonatal diabetes. As the most common cause of PNDM, any neonatal case of diabetes mellitus, in particular among consanguineous parents is a real candidate for molecular analysis of *EIF2AK3* gene. On the other hand, episodes of hepatic failure are the most life threatening complication of this rare syndrome (4). Therefore, early correct recognition and diagnosis is recommended to ensure rapid intervention to prevent hepatic complications.
